# Cervicomedullary motor evoked responses in individuals with severe chronic hemiparesis post-stroke: a feasibility study

**DOI:** 10.3389/fneur.2026.1722620

**Published:** 2026-03-04

**Authors:** Carley L. P. Butler, Aditya Dutt, Carolee J. Winstein, Monica A. Perez, Mary Ellen Stoykov

**Affiliations:** 1 Arms and Hands Lab, Shirley Ryan AbilityLab, Chicago, IL, United States; 2 Department of Physical Medicine and Rehabilitation, Feinberg School of Medicine, Northwestern University, Chicago, IL, United States; 3 Department of Biomedical Engineering, Northwestern University, Evanston, IL, United States; 4 Edward Hines Jr. VA Hospital, Hines, IL, United States; 5 Chicago College of Osteopathic Medicine, Midwestern University, Downers Grove, IL, United States; 6 Division of Biokinesiology and Physical Therapy, Herman Ostrow School of Dentistry, University of Southern California, Los Angeles, CA, United States; 7 Department of Neurology, Keck School of Medicine, University of Southern California, Los Angeles, CA, United States

**Keywords:** cervicomedullary, corticospinal tract, electrical stimulation, hemiparesis, motor evoked potential, stroke

## Abstract

Understanding the neural mechanisms underlying upper limb motor recovery after stroke remains a significant challenge in rehabilitation research. It has been proposed that individuals who show no motor-evoked potential (MEP) response to transcranial magnetic stimulation (TMS) and are thus classified as MEP negative (MEP−) have limited potential for recovery in part due to damage of the corticospinal pathway. In this study, we investigate how individuals categorized as MEP− with TMS respond to stimulation of the corticospinal pathway at a subcortical level. We describe the methodology for eliciting MEPs by using cervicomedullary electrical stimulation (CMEP) in post-stroke individuals with severe upper limb hemiparesis. MEP status (+/−) of the more affected arm was assessed using TMS and cervicomedullary electrical stimulation in stroke survivors with severe upper extremity hemiparesis. While most of the participants were classified as MEP−, all individuals were categorized as CMEP+ in the biceps brachii, extensor carpi radialis, and first dorsal interosseous muscles. Importantly, we report the first testing of CMEPs in a small cohort of individuals with stroke. This technique is feasible in this population and has potential for application in clinical translation settings. Our findings provide a foundation for future studies to replicate and expand upon this approach, enabling the exploration of new hypotheses related to post-stroke rehabilitation and recovery.

## 1 Introduction

Upper extremity (UE) hemiparesis is a common post-stroke disability. The neural mechanisms that enable individuals to regain control of their paretic limb remain poorly understood. There is consensus that the presence or absence of a motor-evoked potential (MEP) elicited by transcranial magnetic stimulation (TMS) over the motor cortex is a useful biomarker for predicting functional recovery in individuals post-stroke in the acute and subacute phases (1). MEP status has also been associated with motor recovery in the chronic phase, although its analytical value is weakened (2). The implication of this classification is that post-stroke individuals without reliable MEPs in the UE (MEP−, i.e., MEPs cannot be elicited even during active muscle contraction and with intensity set to 100% of the maximal stimulator output, MSO) are less likely to benefit from motor training and may lack the capacity for training-induced neuroplasticity.

Unfortunately, there is little research on the neural mechanisms of individuals with moderate to severe hemiparesis. Harris-Love and colleagues investigated functional connectivity using TMS in individuals with moderate to severe impairment (3). It was found that neural changes in response to physical training were optimally reflected in measures of intra- and interhemispheric inhibition. Using TMS, Chelette et al. investigated the long-term cortical reorganization of a single individual with severe post-stroke motor impairment, and they found dynamic changes in the contralesional cortex (4). More recently, studies have shown that post-stroke individuals, categorized as both MEP− and severely impaired, improved in behavioral measures following task-specific training augmented with neuromodulatory techniques (4–6). These findings indicate the need to assess the nervous system in more detail in individuals who are severely impaired and MEP−.

One possible cause of difficulty eliciting MEPs over the primary motor cortex is that TMS is inherently affected by the temporal dispersion of descending volleys, including direct (D) and indirect (I) waves (7). Stimulation of the corticospinal axons more directly, as is done with electrical stimulation of the cervicomedullary junction (CMS), decreases the temporal dispersion of descending volleys increasing the probability of a positive response (8). CMS likely recruits the same corticospinal tract fibers than those activated by TMS by stimulating the primary motor cortex (8, 9). It has been demonstrated that cervicomedullary motor evoked potentials (CMEPs) primarily reflect activation of the corticospinal tract at a subcortical level, as cortical TMS responses were facilitated by the arrival of preceding subcortical CMS volleys and suppressed by collision with antidromic volleys from subcortical CMS (9).

The overall objective of this study was to test residual corticospinal connectivity in individuals with severe post-stroke upper limb impairment by observing the presence or absence of CMEPs, which have been widely examined in control participants (10–15) and individuals with spinal cord injury (16–18). In a previous study, descending connectivity in lower limb muscles was detected in individuals with spinal cord injury by stimulation of the thoracic spine, but in some participants, MEPs could not be elicited using TMS, even at 100% of the MSO (16). Dukipatti and colleagues compared CMEPs in adults with cerebral palsy (CP; with unilateral or bilateral involvement) to control participants and found that diminished CMEP amplitude in the flexor carpi radialis at relatively high levels of stimulation was linked to poor performance on measures of hand function (19). Surprisingly, we found no studies that examined CMEPs in stroke survivors. Therefore, our specific objective was to determine the feasibility of using CMEPs in individuals with moderate to severe stroke-induced upper limb hemiparesis.

This exploratory study will allow translation of a technique previously reported for detecting functional connectivity in the spinal cord injury population to a small cohort of chronic stroke survivors. We also assessed the status of corticospinal connectivity through the presence or absence of MEPs and CMEPs (MEP+/−, CMEP+/−) in three upper limb muscles of the affected arm during voluntary contraction, following administration of the Fugl-Meyer Test of Upper Extremity Function (FMUE) and the Modified Ashworth Scale (20). We hypothesized that CMEPs would be present in individuals with severe post-stroke hemiparesis categorized as MEP−.

## 2 Materials and methods

### 2.1 Recruitment and screening

We recruited 12 individuals: six were identified as MEP− in a previous clinical trial (NCT03517657) and six were individuals with extremely low FMUE scores (≤16). The sample size was determined based on a previous study exploring neural mechanisms in individuals with severe upper limb hemiparesis (3). All participants had previously provided consent via the Clinical Research Registry, enabling contact from researchers at both Shirley Ryan AbilityLab and Northwestern University. All participants gave informed consent to the experimental procedures, which were approved by the local ethics committee at Northwestern University (IRB #: STU00214789) and performed in accordance with the *Declaration of Helsinki*. Inclusion criteria were: (1) Participants who were at least 6 months post-stroke onset from ischemic or hemorrhagic type; (2) severe to moderate upper limb impairment as documented by a FMUE (20) score of ≤35; and (3) categorized as MEP− in at least one of the three muscles tested. Participants from the previously cited clinical trial were MEP− in the extensor carpi radialis (ECR) (NCT03517657). Exclusion criteria were: (1) contraindications to TMS (2); (2) other neurological conditions in addition to stroke; and (3) the presence of severe pain or other upper limb conditions in the affected arm (e.g., recent surgery, orthopedic conditions). Initial screening was conducted via the telephone using a TMS Safety Checklist (2) to exclude individuals who were not appropriate for the study based on safety guidelines or who had other upper limb problems in addition to weakness. The recruitment process is summarized in Supplementary Figure S1. The initial cohort comprised six individuals previously enrolled in the cited clinical trial and classified as MEP− in ECR, all with FMUE scores ≥20. Subsequently, we recruited an additional six individuals with FMUE scores ≤16 who had not undergone prior MEP testing. To confirm MEP status in all participants, TMS was performed in all participants, with eligibility requiring MEP− status in at least one of three muscles tested. All enrolled participants underwent both TMS and CMS. No adverse events were observed during the procedures.

### 2.2 Electromyographic recordings

**Figure 1:**
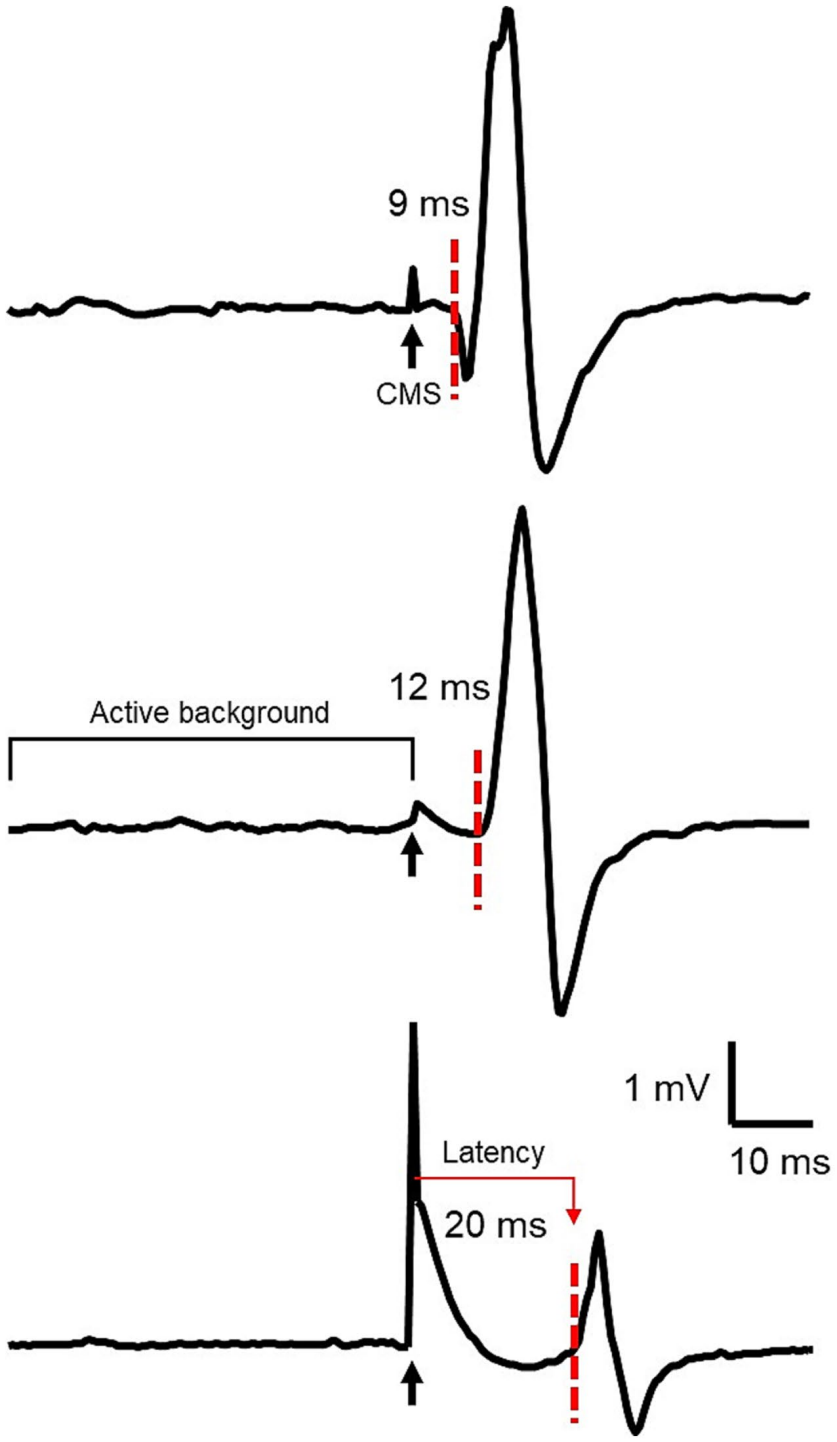
This figure shows the responses to CMS of a representative subject in BB (top), ECR (middle), and FDI (bottom) with EMG in mV (vertical axis) and time in ms (horizontal axis). The black arrows indicate the time of CMS delivery through the stimulus artifact. The red dotted lines indicate response onset. The time between the stimulus and the response is latency, indicated with the red arrow.

**Figure 2:**
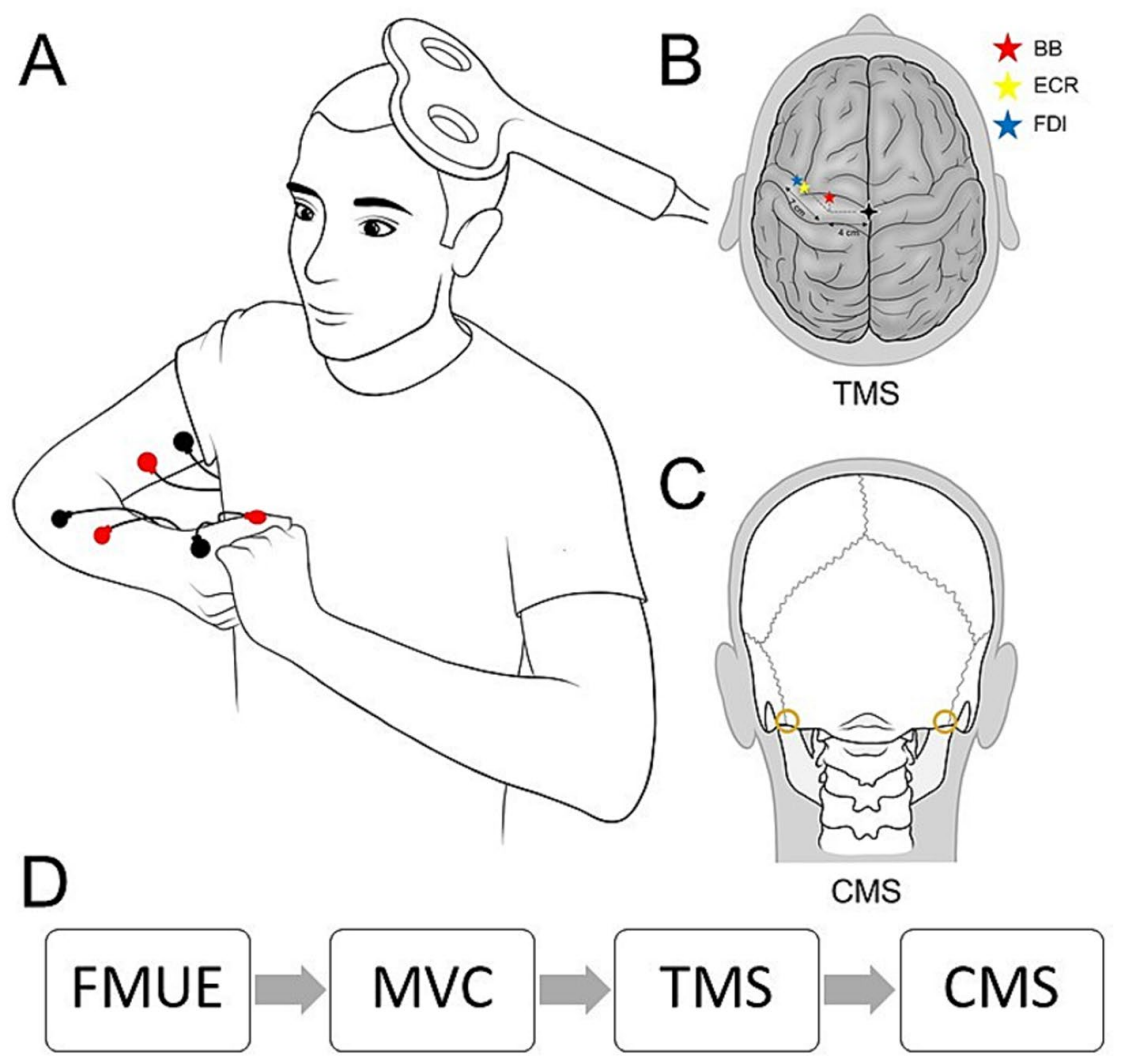
Methods. **(A)** Participants were seated in a customized chair during experimental testing and asked to perform maximal voluntary contraction with hands together as shown. Bipolar surface electrodes were attached to each tested muscle as shown in red and black. TMS was delivered to the contralateral primary motor cortex. **(B)** The approximate stimulation points for each muscle based on previous studies are marked with red (BB), yellow (ECR), and blue (FDI) stars. **(C)** CMS was delivered at the level of the cervicomedullary junction through gold plate electrodes, shown as gold circles. **(D)** The experimental procedure timeline included functional assessment (FMUE), maximum contractions (MVC), TMS, and CMS.

Three UE muscles were selected to represent proximal, intermediate, and distal limb effectors: biceps brachii (BB), ECR, and first dorsal interosseous (FDI) (Figure 1). Participants were seated comfortably in an armchair, and surface EMG recordings were obtained from the affected side. Bipolar Ag-AgCl electrodes (10 mm diameter, 1 cm inter-electrode distance) were placed over the muscle bellies of the BB and ECR. For the FDI, due to its smaller size, the cathode was positioned on the muscle belly and the anode on the interphalangeal joint of the thumb (Figure 2A). EMG signals were amplified by a factor of 100, band-pass filtered (30–2,000 Hz), and digitized at 4 kHz for offline analysis using a CED 1401 interface and Signal 7 software (Cambridge Electronic Design, United Kingdom).

### 2.3 Measurements

#### 2.3.1 MEPs

TMS was delivered over the arm representation of the primary motor cortex from a Magstim 200 stimulator (Magstim Company, Whitland, United Kingdom) through a figure-of-eight coil with a monophasic current waveform. The coil was placed against the scalp at the optimal position for eliciting MEPs in the BB, ECR, and FDI, with the coil held tangentially to the scalp to induce currents in the brain flowing in a posterior–anterior direction (Figure 2A). Minor adjustments of approximately 1 centimeter (7, 21) were made to the coil position to determine the optimal placement for each muscle, starting from the approximate target for each muscle (Figure 2B) and checking in increments around the scalp above the anatomically expected location of the UE representation of the primary motor cortex. Participants wore a cap on which the position of the coil was marked to ensure the stability of TMS across measurements. For individuals who showed no activation at any location on the scalp, the approximate target was used for statistical measures of pre-activation during 10 consecutive stimuli. During TMS at 100% of the MSO, participants were instructed to perform an isometric voluntary contraction of maximal effort into flexion of the elbow, wrist, and finger to increase descending drive to the muscles and promote active facilitation of the response (22). A participant was categorized as MEP + if at least 5 out of 10 consecutive stimuli elicited an active MEP with a peak-to-peak amplitude of at least 100 microvolts above the mean background EMG activity for 100 ms prior to the delivery of the stimulus. MEP responses were further verified by the presence of a cortical silent period following activation, which was noted in the visual feedback of EMG activity (7).

#### 2.3.2 CMEPs

The corticospinal tract was stimulated by a high-voltage electrical current (200 μs duration; DS7R, Digitimer) passed between 2 gold-cup electrodes (GRASS Technologies, Astro Med, Warwick, RI) fixed to the skin behind each mastoid process at the cervicomedullary level (8) (see Figure 2C). A participant was categorized as CMEP+ if at least 5 out of 10 consecutive stimuli elicited a CMEP with a peak-to-peak amplitude of at least 100 microvolts above the mean background EMG activity for 100 ms prior to the delivery of the stimulus.

To identify the active threshold, stimulation was applied during voluntary contraction of (1) the affected upper extremity (UE), (2) the less affected UE, and (3) both UEs simultaneously. Once the threshold intensity was achieved, subsequent CMEPs could generally be elicited without difficulty. After each trial, we ensured participants were comfortable for further stimulation. Previous work by Taylor and Gandevia reported some discomfort during electrical CMS in healthy individuals, attributing it to abrupt contractions of the dorsal neck muscles and backward head movement. To address this, we offered each participant a soft cervical collar for added comfort; some participants opted to use the collar, reporting that it made the procedure feel more secure.

In the bilateral active condition, both arms were positioned according to Figure 2A. The active bilateral condition was selected to maximize descending drive, enabling us to use the lowest possible stimulation intensity required to elicit a response. Previous studies have demonstrated that corticospinal responses are more readily evoked during active muscle contraction in healthy participants. Following the established TMS protocol for the PREP algorithm, participants were instructed to contract their elbow, wrist, and finger flexor muscles with maximal effort in both arms. As such, each electrical CMS session began with the bilateral activation condition, and active BB CMEPs could be elicited with intensities as low as ~100 mA. The first stimulus was delivered at 50 mA during simultaneous active contraction of the arm, wrist, and finger muscles, followed by a second stimulus at 100 mA. Subsequent stimuli were increased in increments of 10–20 mA, depending on participant preference, until the response threshold was reached in all three muscles. Notably, larger responses were observed when the cathode was placed on the affected side. To provide adequate rest and minimize fatigue from repeated contractions, stimulation was delivered at a rate of one per minute, with additional rest breaks as needed. Importantly, we monitored the stimulation to ensure that it was below the intensity required to directly activate the peripheral nerve at the cervical root by ensuring there was no decrease in latency with higher-intensity stimulation. An onset latency decrease of ~2 ms indicates that the response reflects a mixture of pre- and post-synaptic activated motoneurons (8).

#### 2.3.3 Behavior and spasticity

We administered the FMUE (20) and the Modified Ashworth Scale (23) for elbow, wrist, and finger muscles prior to stimulation. The experimental procedure timeline is outlined in Figure 2D.

### 2.4 Analysis

Responses to stimuli from both TMS and CMS were examined to determine latencies and peak-to-peak amplitude values using waveform averages of each stimulation type and muscle at the lowest threshold stimulation levels for BB and ECR (~ 100–150 mA) and FDI (~175–350 mA). Note, for each participant, the threshold for FDI was always higher than the threshold for BB and ECR. To rule out the impact of background contraction on the results of the stimulation, a paired *t*-test was performed to compare active voluntary background EMG 100 ms prior to the stimulation between conditions (TMS vs. CMS).

## 3 Results

### 3.1 Recruitment and screening

Initial outreach was conducted via telephone to individuals known from a previous clinical trial study (NCT03517657). Interested and eligible individuals were consented, and one participant did not complete the experiment due to discomfort. Additional recruitment was conducted for individuals with very severe hemiparesis. Some individuals were uninterested and/or ineligible due to a variety of reasons, including UE pain, contraindications to TMS, and transportation limitations precluding attendance. The total number of eligible participants (*n* = 11) included the initial 5 (from the previous clinical trial) and 6 with very severe hemiparesis (FMUE ≤16) who passed the lab screening. Further details on recruitment and screening process can be found in Supplementary Figure S1. Demographic information as well as means and standard deviations of behavioral measures are presented in Table 1.

**Table 1:** Characteristics of participants.

Subjects	FMUE	Motor assessment scale (Ashworth)			Demographics				
		Elbow flexion	Wrist flexion	Finger flexion	Time post stroke (yr)	Age	Race	Affected side	Gender
P01	24	1+	3	0	19	77	Asian	Left	M
P02	25	1+	3	0	11	68	AA	Left	M
P03	28	1+	1+	1+	6	49	AA	Left	F
P04	16	4	4	3	13	32	AA	Left	F
P05	11	3	3	3	28	76	AA	Right	M
P06	35	1+	3	1	9	56	AA	Left	M
P07	23	1+	3	3	2	53	AA	Left	M
P08	5	1+	2	0	14	63	AA	Left	F
P09	6	4	4	1	7	79	AA	Left	F
P10	9	3	3	2	3	67	AA	Right	M
P11	15	2	2	3	10	66	AA	Left	M
P12 (dropout)	20	2	2	1	13	69	W	Left	M
Mean (SD)	18.1 (9.3)				11.3 (7.1)	62.9 (13.5)			

FMUE, Fugl-Meyer Upper Extremity Test of Function; AA, African American; W, White; M, male; F, female.

### 3.2 Tolerance to CMS

No adverse events occurred. One participant (8.3% drop-out) was uncomfortable during the application of both TMS and electrical CMS. During the latter, the participant reported feeling slight dizziness which subsided 1 min after the stimulation ended. One participant (participant #3) tolerated CMS at no higher than 150 mA (Table 2), while others tolerated stimulation at higher intensities up to 400 mA.

**Table 2:** Motor evoked potential status and cervicomedullary motor evoked potential status in chronic post-stroke participants.

Subjects	Transcranial magnetic stimulation (TMS)			Cervicomedullary stimulation (CMEPs)		
	Biceps	ECR	FDI	Biceps	ECR	FDI
P01	MEP+	MEP−	MEP−	CMEP+	CMEP+	CMEP+
P02	MEP−	MEP+	MEP−	CMEP+	CMEP+	CMEP+
P03	MEP−	MEP−	MEP−	CMEP+	CMEP+	**
P04	MEP−	MEP−	MEP−	CMEP+	CMEP+	CMEP+
P05	MEP−	MEP−	MEP−	CMEP+	CMEP+	CMEP+
P06	MEP−	MEP−	MEP−	CMEP+	CMEP+	CMEP+
P07	MEP−	MEP−	MEP−	CMEP+	CMEP+	CMEP+
P08	MEP−	MEP−	MEP−	CMEP+	CMEP+	CMEP+
P09	MEP−	MEP−	MEP−	CMEP+	CMEP+	CMEP+
P10	MEP−	MEP−	MEP−	CMEP+	CMEP+	CMEP+
P11	MEP−	MEP−	MEP−	CMEP+	CMEP+	CMEP+

MEP, motor evoked potential; CMEP, cervicomedullary motor evoked potential; ECR, extensor carpi radialis; FDI, first dorsal interosseous. **Not tested.

### 3.3 MEP status

We found that two individuals previously categorized as MEP− in the ECR converted to MEP + and elicited a response in one muscle. Participant 1 was classified as MEP + in BB and participant 2, previously categorized as MEP− in the ECR 2 years prior, had now converted to MEP + in ECR. No other participants exhibited reliable MEP responses from TMS and thus, they were classified as MEP− (Table 2).

### 3.4 CMEP status

All 11 participants who tolerated high intensity electrical CMS had measurable responses. Ten out of these 11 had responses in all three muscles and thus were classified as CMEP+. Participant 3 was classified as CMEP+ in BB and ECR, but the FDI was not tested because the participant did not tolerate stimulation above 150 mA due to discomfort (Table 2). CMEPs were large in amplitude and showed expected latency (Figure 1) [8.9 (1.14) ms in BB, 11.67 (1.46) ms in ECR and 19.67 (2.78) ms in FDI] (8, 24).

### 3.5 Background EMG

There was no difference in background EMG activity between the two active stimulus conditions (TMS vs. CMS) for the biceps (*t* = 0.75, *p* = 0.465), ECR (*t* = 0.74, *p* = 0.472), or FDI (*t* = 0.64, *p* = 0.536), indicating that the difference between MEP and CMEP responses was likely unaffected by a difference in muscle activation prior to the stimuli.

## 4 Discussion

### 4.1 Feasibility of CMS

To our knowledge, this exploratory cohort study is the first to demonstrate the feasibility of electrical stimulation at the cervicomedullary junction in post-stroke individuals with severe upper limb hemiparesis. No adverse events occurred. While the majority of our sample had no response to TMS (*n* = 9, 81.8%), they exhibited robust responses to electrical CMS across three muscles of the arm. One participant did not tolerate electrical CMS and did not complete the experiment. All other participants (*n* = 11, 91.7%) responded well to the stimulation. Cervicomedullary stimulation can also be done via magnetic stimulation with a special coil, and some may prefer that method (25). Stimulation levels used in this study were higher than in a previous study (19) in adult participants living with CP, where CMEPs were elicited in the flexor carpi radialis with intensities between 60 and 95 mA. However, these participants were able to handle objects (with or without difficulty) according to the Manual Abilities Classification System (26), showing better hand function than the stroke survivors tested here. It is likely that higher intensity stimulation is needed for more impaired individuals.

### 4.2 Corticospinal connectivity

We found that two moderately impaired participants were MEP + in a single muscle and MEP− in the other two muscles tested. Our findings are in agreement with previous findings showing that 40% of moderately impaired participants were likely to be MEP− in one hand muscle and MEP + in another (27). Thus, testing and labeling a muscle as MEP+/− may be more suitable than labeling a participant from the status of one muscle.

Electrical CMS elicit responses following stimulation of corticospinal axons, providing a straightforward method to evaluate corticospinal tract connectivity during and after tasks requiring voluntary movement; it may also test the efficiency of descending control and its modulation under variable circumstances (8). Indeed, measurement of CMEPs may provide a method of probing the subcortical effects of motor learning. Stimulating the corticospinal tract cortically and subcortically can provide an important comparison for interpreting changes in individuals with motor impairment and may lead to varying methodologies and treatment possibilities to optimize recovery post-stroke. Limiting exploration of neural plasticity to the cortex, as done with TMS, reduces the ability to comprehend the full potential of the damaged nervous system (9). Measures of subcortical and spinal plasticity are appropriate to document treatment-induced changes. Thus, electrical CMS may be a valuable technique for testing the effects of neuromodulation that aims to examine corticospinal contributions. With the increase in spinal and cerebellar neuromodulation protocols (28–34), it is in the interest of the neural recovery research community to differentiate cortical, subcortical, and spinal contributions to motor learning, including behavioral changes following interventional protocols.

### 4.3 Limitations

Limitations of this study include its exploratory nature and the small number of participants. One physiological limitation is that stimulation of other tracts may occur during CMS; however, this can also occur with high intensity TMS. In a previous collision study, MEPs were attenuated when cortical stimulation was preceded by CMS, indicating that CMEPs and MEPs are likely to travel along the same pathway (9).

## 5 Conclusion

(1) CMS is effective and tolerated in a small cohort of chronic stroke survivors with severe hemiparesis FMUE Mean/SD 18.1 (9.3), and 91.7% of the cohort tested as CMEP+ using electrical CMS in three UE muscles. All individuals were able to achieve CMEP+ status in at least two UE muscles tested. This stimulation technique may provide a more sensitive method to assess corticospinal excitability in stroke survivors than TMS. These findings are a useful starting point for neurophysiological investigation into mechanisms of recovery post-stroke, and the next steps should include a larger and more diverse sample size.

## Data Availability

The raw data supporting the conclusions of this article will be made available by the authors, without undue reservation.

## Supplementary material

SUPPLEMENTARY FIGURE S1 This figure shows a flowchart of the systematic recruitment process, including inclusions and exclusions of participants and the resulting 11 subjects for the experiment.
