# Supplementary figures and images for: Cervicomedullary motor evoked responses in individuals with severe chronic hemiparesis post-stroke: a feasibility study
Source: Front Neurol. 2026 Mar 4;17:1722620. doi: 10.3389/fneur.2026.1722620 (PMC12996075; doi:10.3389/fneur.2026.1722620)

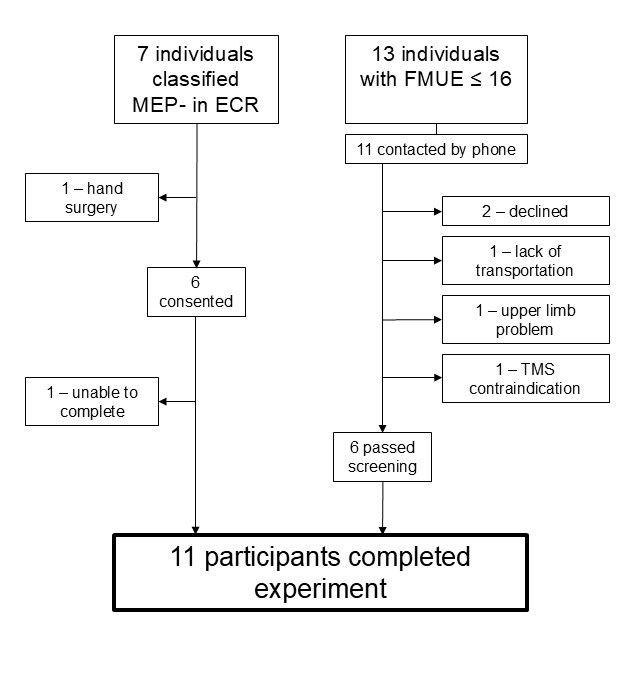

Supplement: SUPPLEMENTARY FIGURE S1 — This figure shows a flowchart of the systematic recruitment process, including inclusions and exclusions of participants and the resulting 11 subjects for the experiment. [file Image_1.jpeg]
